# Adherence to Antimalarial Drug Therapy among Vivax Malaria Patients in Northern Thailand

**DOI:** 10.3329/jhpn.v27i1.3313

**Published:** 2009-02

**Authors:** Nardlada Khantikul, Piyarat Butraporn, Han S. Kim, Somjai Leemingsawat, M.A. Sandra B. Tempongko, Wannapa Suwonkerd

**Affiliations:** ^1^ Faculty of Tropical Medicine, Mahidol University, 420/6 Rajvithi Road, Rajdhevi District, Bangkok 10400, Thailand; ^2^ Department of Family and Preventive Medicine, University of Utah, 375 Chipeta Way, Suite A, Salt Lake City, Utah 84108, USA; ^3^ SEAMEO Office Network Bangkok, 420/6 Rajvithi Road, Rajdhevi District, Bangkok 10400, Thailand; ^4^ Office of Disease Prevention and Control, 10 Chiang Mai, 447 Lamphun Road, Muang District, Chiang Mai 50200, Thailand

**Keywords:** Antimalarials, Chloroquine, Drug adherence, Drug therapy, Primaquine, Malaria, Retrospective studies, Vivax malaria, Thailand

## Abstract

Vivax malaria is a significant cause of morbidity due to malaria in northern Thailand, accounting for approximately 50% of all malaria cases. The objective of this study was to determine the behavioural factors associated with adherence to the standard 14-day course of chloroquine and primaquine, prescribed from malaria clinics, among patients with vivax malaria. A retrospective study was conducted among 206 patients living in Muang and Mae Sa Riang districts of Mae Hon Son province in northern Thailand. Data on adherence and potential behavioural factors relating to adherence were collected using a structured interviewer-administered questionnaire and supplemented with qualitative data from focus-group interviews. The results indicated that 76.21% of the 206 patients with vivax malaria did not complete the medication course. The adherence of the patients was associated with knowledge scores of malaria (adjusted odds ratio [AOR]=2.2, 95% confidence interval [CI] 1.1-4.5) and accessing drug prescription scores (AOR=5.6, 95% CI 2.13-15.3). Therefore, further effort is needed to educate patients with vivax malaria on knowledge of malaria and its treatment with simple health messages and encourage them to adhere to their treatment.

## INTRODUCTION

*Plasmodium vivax* is a significant cause of morbidity due to malaria in northern Thailand which has a population of 4.74 million with mixed ethnic Thai and hill-tribe minority groups. Early in the new millennium, the relative prevalence of *P. falciparum* and *P. vivax* came close to equilibrium, and recently, *P. vivax* became the leading species. In 2005, 2,699 microscopically-confirmed cases were reported, of which 46% were due to *P. falciparum,* and 54% were due to *P. vivax* (1). Malaria due to *P. vivax* has placed the huge burden on health in northern Thailand. In provinces on the Thai-Myanmar border where the transmission of malaria is high, the proportion of *P. vivax*-associated cases has increased slightly over time. Transmission is closely associated with forest locations where there are abundant breeding sites of the vectors of malarial parasites (2), various occupational factors that encourage movement of population or influx of refugees, socioeconomic factors affecting malaria (3,4), and high degree of drug resistance (5).

The Mae Hong Son province is located on the Thai-Myanmar border in northern Thailand. Surveillance reports indicate that malaria has become endemic throughout the Mae Hong Son province. The incidence rates of malaria in the province were 24.4, 14.2, 10.04, 8.52, and 8.32 per 1,000 people during 2001-2005 respectively (1). These incidence rates are significantly higher than that in the national malaria-control programme goal of lower than 1 per 1,000 people (6).

Prompt and effective treatment of malaria remains a challenge for the malaria-control programmes (7). Case management is one of the cornerstones of malaria control in this part of the world, with the goal to reduce morbidity and mortality through prompt diagnosis and effective management of acute clinical episodes with antimalarial drugs (8). Chloroquine and primaquine are still effective for treating patients with vivax malaria in Thailand (9,10). Although drug resistance in *P. vivax* has not yet been reported in Thailand (11), accurate assessment of adherence behaviour is necessary for effective and efficient treatment (12). However, there are a few systematic studies on adherence of patients to antivivax malaria medications. Therefore, we conducted a retrospective study to define the factors influencing the adherence and non-adherence to drug treatment in patients with vivax malaria. The results from this research will be used for implementing a health-promotion programme to enhance adherence to antivivax malaria drugs.

## MATERIALS AND METHODS

### Study site

This retrospective study was conducted during May-September 2006 in Muang and Mae Sa Riang districts, Mae Hong Son province of north Thailand. The Mae Sa Riang district is located 190 km south of Muang district. These areas are of mixed ethnic groups that include Thai, Karen, and Shan (Thai Yai) and are similar in culture, demographics, and natural environment. The settings were selected because of high transmission of vivax malaria.

### Selection of sample

The initial sample comprised all records of 379 vivax malaria patients who received treatment from three malaria clinics in Muang district and two in Mae Sa Riang district during 1 January 2005–31 July 2006; cases were diagnosed by thick blood smear examinations and had single infection with *P. vivax*. Of the 379 patients with vivax malaria, 206 were eligible for inclusion in the study. The remaining 173 cases were excluded because 135 were not residents, 12 had mixed vivax and falciparum infections, 16 were treated at hospitals, and 10 refused to participate in the study. Focus-group discussions (FGDs) were conducted in September 2006 to collect more information and triangulate with quantitative data. We conducted FGDs four times with the participants who were randomly selected from those of 206 interviewed patients after the collection of quantitative data. We sampled them from the list of our patients and then made an appointment. Village health volunteers assisted us to call them to a meeting place. The Mae Sa Riang district had two FGDs: the first one had seven adherent patients, and the second one had eight non-adherent patients. The Muang district conducted two group discussions: the first one was conducted with eight adherent patients, and the other one with seven non-adherent patients.

### Training of interviewers

Four skilled intervierwers were trained to administer the structured questionnaire, the use of informed consent forms, procedures of questioning the participants, and interacting with four interpreters. All the four interpreters received training at the same time with the interviewers while the trainer had taught them some simple scientific terms and cross-checked the meaning between the both, such as symptoms of malaria, primaquine, chloroquine, etc. One interpreter accompanied an interviewer to visit homes of patients throughout the study. All the interviewers were able to listen terms/dialects of native languages; however, they might not speak the native language as well as did the interpreters.

### Instruments

We used an interviewer-administered structured questionnaire for data collection and developed focus-group guidelines (13) to collect qualitative data. The questionnaire was designed to elicit information from participants on behaviours relating to adherence to antimalarial medicines prescribed by the malaria clinic. The structured questionnaire was pretested in a distant similar geographical district in Chiang Dao, Chiang Mai province. The questionnaire consisted of questions relating to general knowledge of malaria, perception towards susceptibility and severity of malaria, benefit from and barrier to treatment, and an access to information on antimalarial medications and adherence to drug. Scores on knowledge about malaria (14) were assessed using 15 questions (alpha reliabili-ty=0.82) that asked the participants about aetiology of malaria, breeding places of malaria vectors, and prevention and control of malaria. The access to information on malaria-medication scores (8) was calculated using eight questions (alpha reliability=0.61). Each question was scored with 1 for a correct answer or 0 for an incorrect one. Therefore, the knowledge score ranged from 0 to 15, and the access to information on antimalarial medication scores ranged from 0 to 8. The knowledge of malaria and the access to information on antimalarial medication scores were categorized into two levels using cut-off points at mean scores. Perception on malaria treatment referred to 15 questions (alpha reliability=0.64), including perceived susceptibility which refers to an individual perceiving her/himself vulnerable to contract the disease, perceived seriousness which means an individual's belief that malaria is serious, perceived benefit from treatment which refers to the degree to which an individual believes the effectiveness of medications, and perceived barriers associated with adherence, such as forgetting to take the medicine, getting side-effects, and lack of access to antimalarial treatment. The malaria-perception scale-related questions use a Likert-type scale (15). The possible range of scores for the perception scale was 15 to 45, and this scale was also dichotomized at the mean scores as mentioned above. We also collected qualitative data through FGDs with adherent and non-adherent participants from the two districts. We defined adherence as a completion of the prescribed medication regimen without deviation. The drug regimen consisted of chloroquine 25 mg base/kg body-weight per day taken for three days and primaquine 0.25 mg/kg for 14 days.

### Data collection

An interviewer administered the structured questionnaire at the patient's house. We collected name, address, and history of malaria of each eligible patient from the patient-records that would bring us to the patient's home. When visiting for interview, 206 patients participated after 10 patients refused the consent. A supervisory team monitored the interviewers and checked the questionnaire for consistency. In the FGDs, the principal investigator who acted as the moderator worked with two assistants sitting in circle with the participants. During about one hour of discussion, information was taped and noted.

### Statistical analyses

The Stata software (version 8.0) (Stata Corporation, College Station, TX, USA) was used for all statistical analyses. Chi-square tests were used for identifying variables associated with adherence to drug, including knowledge of malaria, perception of malaria, drug-taking behaviour, healthcare service, and social support. The variables that were significantly associated with adherence to drug in bivariate analyses (p<0.05) were selected for further multivariate analyses using unconditional logistic regression to assess confounding and interactions between the determinants. Odds ratios and 95% confidence intervals were used for quantifying associations. All statistics were two-sided tests with a significance level of alpha=0.05. Data from the FGDs were analyzed using a deduction method.

### Ethical considerations

The Ethical Committee of the Mahidol University reviewed and approved the procedures of this study. Patients were informed about the purpose of the study, and the participants provided written consent before participating in the interview process.

## RESULTS

### Demographics

Of the 206 patients with vivax malaria, 133 (64.5%) were male and 155 (75.2%) were non-Thai comprising Karen (34.0%), Thai Yai (27.7%), and others (3.4%); 76.2% of the patients with vivax malaria did not complete the medication course whereas 23.8% adhered to it (Table 1). Languages used included Thai, Karen, and Thai Yai. Over 40% had never attended any school. The median age of the study participants was 36 (range 11-88) years, and the median family-size was 4 (range 1-7). There was some variability across the study sites in the median monthly income. The lowest monthly income was zero, the median was 1,350 Baht (US$ 39), and the highest was 24,000 Baht (US$ 705). The mean number of years of residency at the respondent's current location was 40 years. About 64.0% were farmers who worked with forestry-related jobs.

**Table 1.** Associations between demographic characteristics of patients with vivax malaria and adherence to drug, Mae Hong Son province, northern Thailand, 2006

Demographic characteristics	Non-adherent (76.2%)		Adherent (23.8%)		Chi-square
	No.	%	No.	%	(p value)
Gender					
Male	98	62.4	35	71.4	0.249
Female	59	37.6	14	28.6	
Age-group (years)					
11-35	79	50.3	20	40.8	0.245
36-88	78	49.7	29	59.2	
Ethnicity					
Thai	39	24.8	12	24.5	0.960
Non-Thai	118	75.2	37	75.5	
Occupation					
Not forestry-related∗	62	39.5	12	24.5	0.056
Forestry-related	95	60.5	37	75.5	
Educational level					
No schooling	71	45.2	17	34.7	0.193
Primary schooling and above	86	54.8	32	65.3	
Income per month (Baht)					
<1,000	74	47.1	24	49.0	0.821
>1,000	83	52.9	25	51.0	
Number of family members					
<5	103	65.6	29	59.2	0.413
>5	54	34.4	20	40.8	

∗Includes students, housewives, government officials, employees, and unemployed

### Knowledge

There was a significant difference in knowledge on malaria between the adherent and the non-adherent group (Table 2). Participants who adhered to their malaria medication were more likely than non-adherent participants to correctly answer that mosquito bites are a cause leading to malaria (p=0.015), that one cannot contract malaria by drinking water from a stream in the forest (p=0.001), that malaria vectors breed in a slow-running stream (p=0.001) and in animal footprints in the forest (p=0.008), and that sleeping under a mosquito net (p=0.011) and using mosquito-repellent (p=0.040) can prevent malaria. Both adherent and non-adherent groups recognized that headaches, fever, and shivering were common signs and symptoms of malaria.

**Table 2.** Comparison of general knowledge of malaria and adherence to drug among patients with vivax malaria, Mae Hong Son province, 2006

Knowledge of malaria	Number that answered correctly				
	Non-adherent		Adherent		Chi-square
	No.	%	No.	%	(p value)
Malaria transmission					
Mosquito bites cause malaria	110	70.1	43	87.8	0.015
Drinking raw water from a stream in the forest is not a risk	35	22.3	25	51.0	0.001
Breeding place for malaria vectors					
Vectors breed in slow-running streams	45	28.7	27	55.1	0.001
Vectors breed in animal footprints	81	51.6	36	73.5	0.008
Protective methods against malaria					
Sleeping under mosquito net	104	66.2	42	85.7	0.011
Using mosquito-repellent	96	61.1	38	77.6	0.040
Signs and symptoms of malaria					
Headache	133	84.7	45	91.8	0.241
Fever	138	87.9	47	95.5	0.173
Shivering	130	82.8	43	87.8	0.507

### Perceptions

The figure illustrates that the overall mean perception score differed significantly between the adherent and the non-adherent patients (p=0.011). Those with good perception were 2.2 times (95% CI 1.1-4.9) more likely to adhere to antimalarial medications than those with poor perception. After stratifying the results into perception subcategories, perceived susceptibility and perceived severity of malaria were not associated with adherence. However, those with good perceived benefit from antimalarial medications were 3.3 times (95% CI 1.6-6.7) more likely to adhere to the medications than those with poor perceived benefits. Similarly, those with good perceived barriers to antimalarial medications were 1.91 times (95% CI 1.0-3.9) more likely to adhere to the medications than those with poor-perceived barriers.

**Fig.**
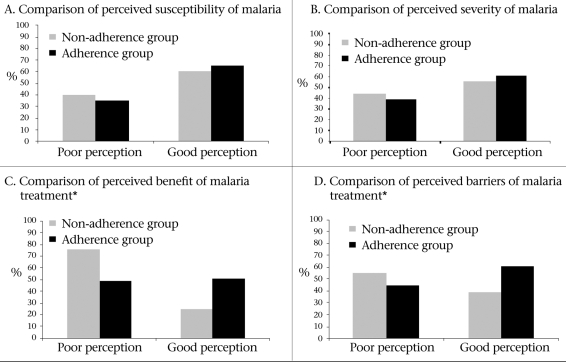
Comparisons between poor and good perceptions in term of: (A) Perceived susceptibility of malaria, (B) Perceived severity of malaria, (C) Perceived benefit of malaria treatment, and (D) Perceived barriers to malaria treatment among patients with vivax malaria in Mae Hong Son province, 2006 (∗indicates p<0.05)

### Access

Table 3 describes access to information received on antimalarial medications by patients with vivax malaria after visiting malaria clinics, stratified by type of adherence. We asked whether the patients could recall and explain the drugs received from the malaria officers. The proportions of 75.5% of the adherent patients (n=37) higher than 55.4% of the non-adherent patients (n=87) stated taking chloroquine and primaquine for full 14 days (p=0.013), could recall the colour of the pills (83.7% vs 52.2%, p=0.000), and could clearly understand and recall the labels and instructions (81.6% vs 57.3%, p=0.002). For other questions on access to information, the proportions were statistically similar. Further analysis of the summary data (Table 4) revealed that those with high access to information on antimalarial medication scores were 5.6 times (95% CI 2.0-18.9) more likely to adhere to treatment than those with low access scores.

**Table 3.** Comparisons of access to information on antimalarial medications and adherence to drug among patients with vivax malaria, Mae Hong Son province, 2006

Determinant	Non-adherent		Adherent		p value
	No.	%	No.	%	
Stated taking chloroquine and primaquine for full 14 days	87	55.4	37	75.5	0.013
Could recall colour of antimalarial pills correctly	82	52.2	41	83.7	0.000
Received drug package from malaria clinics correctly	124	79.0	42	85.7	0.408
Took the first dose of antimalarial drug immediately after diagnosis	122	77.7	38	77.6	1.000
Clearly understood drug label and instructions	90	57.3	40	81.6	0.002
Malaria officer clearly explained how to complete treatment for malaria	130	82.8	45	91.8	0.169
Understood all details	121	77.1	43	87.8	0.154
Took drug after meal to decrease side-effects of treatment for malaria	98	62.4	35	71.4	0.306

**Table 4.** Logistic regression analysis to identify determinants for knowledge of malaria, perception of malaria, access to information on prescription, and adherence to drug among patients with vivax malaria

Variable	Unadjusted		Adjusted	
	OR	95% CI	OR	95% CI
Knowledge scores				
Poor (<10)	1.0		1.0	
Good (10-15)	2.1	1.0-4.3	2.2	1.1-4.5
Perception scores				
Low (<38)	1.0		1.0	
High (38-45)	2.3	1.1-4.9	1.9	0.9-3.8
Access to drug prescription scores			
Poor (<6)	1.0		1.0	
Good (6-8)	5.6	2.0-18.9	5.6	2.1-15.3

CI=Confidence interval; OR=Odds ratio

### Adherence

After adjusting for demographic factors, knowledge scores, perception scores, and access to information on antimalarial medication scores, adherence of patients was significantly associated with knowledge of malaria scores (adjusted OR=2.2, 95% CI 1.1-4.5), and access to information on antimalarial medication scores (AOR=5.6, 95% CI 2.1-15.3). Patients having higher or equal to 38 perception scores were 1.91 times (AOR=1.9, 95% CI 0.9-3.8) more likely to be adherent than those with lower than 38 perception scores; these results were not statistically significant (Table 4).

### Focus-group discussions

The qualitative data from FGDs in two districts of the province fostered the findings from questionnaire interviews for perceptions of benefits and barriers to the medications. The perceived benefits appeared to be related to confidence in drug-effectiveness among the adherent patients. These cases perceived barriers to treatment when they got sick from side-effect of chloroquine; however, they were not feared to fight against it, for example, trying to take drugs before bed or taking drugs immediately after a meal. A female respondent stated, “Medication course takes so long to finish, I got vomiting only in the first three days but I tried to finish all the drugs.” A man who perceived that going to treatment at the clinic was time-consuming, stated, “I have to go for treatment anyway, even I miss my job for one day.”

## DISCUSSION

*P. vivax*-associated infection has placed huge burdens on the health, longevity, and general prosperity in large segments of the human population (16). Adherence to antimalarial medications remains an important component of malaria control. According to the policy of the Thai National Malaria Control Programme, all microscopically-confirmed cases of vivax malaria receive prompt treatment following the National Drug Guidelines of the Ministry of Public Health (6). The ability of people to follow a given treatment regimen has been studied for some time. ‘Compliance', ‘concordance', and ‘adherence' have all been used for describing their concepts (17). Adherence is difficult to measure as the techniques to measure adherence have drawbacks (18). In this study, adherence behaviour was assessed using self-report measures that have been standardized, validated, and well-accepted in the adherence literature (19). Patients were classified as ‘adhering' if they followed the medication instructions exactly by taking the standard 14-day course of chloroquine (25 mg base/kg for 3 days) and primaquine (0.25 mg/kg daily for 14 days) (20). However, the large majority (76.2%) of patients with vivax malaria in this study were classified as ‘non-adhering'. The consequences of non-adherence to antimalarial medication may not only be dangerous for the health of the patient, but may also dramatically increase the financial cost for public-health services (11).

The demographic factors have not been shown as predictors of adherence. This finding was similar to the study of Haynes (21) that found no relationship between adherence and race, sex, educational experience, intelligence, marital status, occupational status, income, and ethnic or cultural background and was also similar to results reported by Simsek (22). However, in bivariate analyses, only those who had forestry-related occupations were more likely to adhere to treatment than those not involved in forestry-related occupations, with a p value that closely approaches statistical significance (p=0.056). In contrast, the participants not involved in forestry-related occupations from FGDs indicated that, since they worked in or near areas with high transmission of malaria, they wanted to avoid having chronic malaria which may prevent them from working; so, they had a greater incentive to complete the medications for malaria to avoid missed work days.

Results of this study also suggest that general knowledge of malaria may be associated with adherence to drug. Symptoms commonly relating to malaria were fever, headache, and shivering, specifically fever that reappears with certain regularity. Both adherent and non-adherent participants were equally knowledgeable about the signs and symptoms of malaria. Prior studies have shown that populations living in malarial-prone areas are generally knowledgeable about the signs and symptoms of malaria. Guatemala and Turkey reported recognition of symptoms of malaria in more than 80% of the study population (22,23). Outside of signs and symptoms of malaria, there is, however, a fair amount of misinformation on aetiology of malaria. For example, a significant number of respondents (p<0.05) reported that malaria can be contracted by drinking untreated water from streams, ponds, or groundwater in the forest. Only 50% of the adherent group knew breeding places of malaria vectors and protective measures against malaria (24). Nevertheless, those who were adherent to medication for vivax malaria were significantly more knowledgeable in aetiology of malaria than those who were non-adherent, indicating that better education in aetiology of malaria may increase adherence to treatment (AOR=2.24, 95% CI 1.11-4.50). The information from FGDs supported that the participants did not know about drug of treatment, action of drug, the way how to take it correctly, relapsing fever, and what are chloroquine and primaquine for. They might not be aware of the hazards of not adhering to the treatment of malaria.

Perceived benefits from effectiveness of medication and barriers of forgetting to take medications were related to beliefs about receiving treatment at the malaria clinic and how treatment would affect the well-being of an individual. Adherent and non-adherent patients answered that they went to malaria clinics for medical attention when ill. The malaria clinics in this study are located closely to their houses and accessible on the well-paved roads. In terms of the Health Belief Model (25), cues to action from sickness of their relatives are the factors that might encourage an individual to act in the face of their perception of the severity of vivax malaria. In our study, adherence was significantly associated with perceived benefit from and perceived barrier to malaria-treatment scores (p<0.05). The participants had confidence with treatment of malaria from the malaria clinic. This may provide a further path of action to increase drug-adherence behaviour among patients with vivax malaria.

Further, more information on access to antimalarial medications among patients with vivax malaria, after visiting malaria clinics, was gathered in focus groups. We asked whether the patients could describe the treatment for malaria and what information they received from malaria officers. The adherent group could recall the number of days to complete taking chloroquine and primaquine better than those in the non-adherent group. Atreja *et al*. noted using a multidisciplinary approach with the ‘SIMPLE strategy' (S=Simplifying regimen characteristics, I=Imparting knowledge, M=Modifying patients' beliefs, P=Patients' communication, L=Leaving the bias, and E=Evaluating adherence in the context of a healthcare team and system-related factors) to enhance drug adherence (26). The problem of poor adherence may be overcome with simple health messages even when the majority of individuals are illiterate and lack formal education (27).

In northern Thailand, when medication is administered to patients at malaria clinics, verbal instructions are given, and little written instructions are printed on the 4-5 plastic bags that contain the actual drugs. These bags containing drugs are sometimes divided according to meals. However, our study indicates that information, education, and communication (IEC) materials available to patients are not used at malaria clinics. One patient in the non-adherent group stated, “I confused with several bags given from malaria clinic. I could not understand how to take antimalarial prescriptions after I went home. I could not remember which medicines should be taken and when.” Another one said, “When I feel better, I always forget to take antimalarial drugs on time. I remembered that I did not finish antimalarial drugs. I did not know what happened to me when I did not finish all medicines.” Rates of low adherence may indicate that instructions provided to patients are too complicated for them to understand, and the language used was a barrier for some patients, particularly among ethnic minority groups. Even among patients who speak Thai, literacy may be an issue, similar to other studies that have reported difficulties due to limited literacy among patients and their inability to read written instructions (28,29). Furthermore, qualitative data from focus-group interviews suggest that patients were not able to understand or remember the instructions and suggestions from malaria officers because patients were so ill when they were receiving medications, regardless of literacy or language used. Moreover, these may be overcome by tailoring education to the patient's level of understanding for instructions about medicine-use. The next step is, therefore, to look for different models of IEC programmes for these target groups.

In interviews with subjects, we found that 55.8% of the patients with vivax malaria were satisfied with the healthcare services from the malaria clinics. Critical to patients' adherence is good communication between malaria officers and patients. Healthcare providers play a unique and important role in assisting healthy behaviour changes among patients (30-32). The role of healthcare providers should go beyond case-investigations and prompt treatment to ensure that people are receiving the effective drug regimen to treat malaria, and interventions that aid people in taking the correct treatment will help maximize their effectiveness.

This study had both limitations and strengths. The strength of this study is that, to date, this is the only study in Thailand that looks at social factors for adherence to treatment for vivax malaria. This study also includes a sufficiently large number of minority subjects with an adequate number of participants. Furthermore, both qualitative and quantitative data were collected. Since this study is retrospective and the participants were asked to recall actions that occurred up to a year in the past, recall bias is a potential issue. Those who did not adhere to the treatments were more likely to incorrectly recall their treatments. Also, those who did not adhere to the treatments may be less likely to participate; so, there is potential for some participation bias. Finally, because of various languages spoken by the study participants, there may be information biases due to interpretation issues, although any bias due to interpretation is most likely non-differential.

The success or failure of any public-health programme is largely determined by effective use of services offered to the public. This principle is especially relevant in treating malaria because the prompt use of effective drugs greatly reduces the incidence of severe and complicated disease (12). Adherence to antimalarial medication has been correlated to knowledge of malaria, access to information on medication for malaria, perceived benefit from medication, and perceived barriers to treatment. Non-adherent patients with vivax malaria are at an increasing risk of consequent relapses, increasing morbidity due to malaria, and rising overall cost of treatment. However, changing human behaviours to increase adherence can be difficult, and to maintain these behavioural changes is even more challenging. A sustainable tool to overcome these challenges is the use of IEC strategies. The results of this study have given us insights into the social factors associated with adherence to antimalarial medications and can, therefore, provide baseline data for planning new strategies to improving adherence to antimalarial medications. Specifically, we can use these data to develop tailored educational materials oriented towards strengthening knowledge of malaria among at-risk populations. Finally, to overcome behavioural barriers to adherence to antimalarial medications, improved IEC between healthcare providers and patients are critical to enhance the ability of patients to follow the medication regimen.

## ACKNOWLEDGEMENTS

This study received financial support from the Royal Golden Jubilee PhD Programme, Thailand Research Fund (Grant No. PHD/0232/2547) and the WHO/SEARO in collaboration with UNDP/World Bank (Ref. file T/16/72/83).
